# Errors in radiation oncology: A study in pathways and dosimetric impact

**DOI:** 10.1120/jacmp.v6i3.2105

**Published:** 2005-08-17

**Authors:** Eric E. Klein, Robert E. Drzymala, James A. Purdy, Jeff Michalski

**Affiliations:** ^1^ Department of Radiation Oncology Washington University School of Medicine St. Louis Missouri 63110 U.S.A.

**Keywords:** radiotherapy, errors, record and verify, error analysis

## Abstract

As complexity for treating patients increases, so does the risk of error. Some publications have suggested that record and verify (R&V) systems may contribute in propagating errors. Direct data transfer has the potential to eliminate most, but not all, errors. And although the dosimetric consequences may be obvious in some cases, a detailed study does not exist. In this effort, we examined potential errors in terms of scenarios, pathways of occurrence, and dosimetry. Our goal was to prioritize error prevention according to likelihood of event and dosimetric impact. For conventional photon treatments, we investigated errors of incorrect source‐to‐surface distance (SSD), energy, omitted wedge (physical, dynamic, or universal) or compensating filter, incorrect wedge or compensating filter orientation, improper rotational rate for arc therapy, and geometrical misses due to incorrect gantry, collimator or table angle, reversed field settings, and setup errors. For electron beam therapy, errors investigated included incorrect energy, incorrect SSD, along with geometric misses. For special procedures we examined errors for total body irradiation (TBI, incorrect field size, dose rate, treatment distance) and LINAC radiosurgery (incorrect collimation setting, incorrect rotational parameters). Likelihood of error was determined and subsequently rated according to our history of detecting such errors. Dosimetric evaluation was conducted by using dosimetric data, treatment plans, or measurements. We found geometric misses to have the highest error probability. They most often occurred due to improper setup via coordinate shift errors or incorrect field shaping. The dosimetric impact is unique for each case and depends on the proportion of fields in error and volume mistreated. These errors were short‐lived due to rapid detection via port films. The most significant dosimetric error was related to a reversed wedge direction. This may occur due to incorrect collimator angle or wedge orientation. For parallel‐opposed 60° wedge fields, this error could be as high as 80% to a point off‐axis. Other examples of dosimetric impact included the following: SSD, ~2%/cm for photons or electrons; photon energy (6 MV vs. 18 MV), on average 16% depending on depth, electron energy, ~0.5cm of depth coverage per MeV (mega‐electron volt). Of these examples, incorrect distances were most likely but rapidly detected by in vivo dosimetry. Errors were categorized by occurrence rate, methods and timing of detection, longevity, and dosimetric impact. Solutions were devised according to these criteria. To date, no one has studied the dosimetric impact of global errors in radiation oncology. Although there is heightened awareness that with increased use of ancillary devices and automation, there must be a parallel increase in quality check systems and processes, errors do and will continue to occur. This study has helped us identify and prioritize potential errors in our clinic according to frequency and dosimetric impact. For example, to reduce the use of an incorrect wedge direction, our clinic employs off‐axis in vivo dosimetry. To avoid a treatment distance setup error, we use both vertical table settings and optical distance indicator (ODI) values to properly set up fields. As R&V systems become more automated, more accurate and efficient data transfer will occur. This will require further analysis. Finally, we have begun examining potential intensity‐modulated radiation therapy (IMRT) errors according to the same criteria.

PACS numbers: 87.53.Xd, 87.53.St

## I. INTRODUCTION

As the complexity for planning and treating radiation oncology patients increases, so does the potential of error. This is partially due to the increased demand for ancillary devices and the introduction of new treatment procedures and techniques. This advancement has not necessarily come simultaneously with an increase in verification capability. In fact, there has been a simultaneous drive to expand automation, attributed to an increase in the number of daily treatment fields. Although the use of record and verify (R&V) systems has increased, some authors^(^ ^1^ ^–^ ^5^ ^)^ have suggested that these devices may contribute in propagating errors, since these systems are frequently utilized to enhance efficiency rather than as quality assurance systems. However, with the recent advent of digital data import from treatment‐planning systems and automated treatment setups, errors associated with manual data entry and setups will be reduced.^(^ ^6^ ^)^ Therefore, it is vital that processes and quality assurance procedures in any radiotherapy department adapt to the new electronic environments so that R&V systems diminish error propagation rather than increase it.

There are several detailed reports on error analysis^(^ ^7^ ^–^ ^9^ ^)^ in radiation oncology in terms of how errors occur, but a detailed analysis of the dosimetric consequences does not exist. In this study, we examined potential errors in terms of different scenarios, pathways of error occurrence, and the subsequent dosimetric impact. Our goal is to prioritize error prevention according to likelihood of an event and its dosimetric impact.

## II. MATERIALS AND METHODS

Our facility treats approximately 2000 patients per year on seven LINACs, each equipped with an R&V system (Varis v1.4g, Varian Oncology Systems or Clinical Desktop v4.10, Elekta Oncology). Over a 30‐month time period, we initiated 3964 courses of therapy. It must be noted that neither of these systems nor the various radiotherapy treatment‐planning systems at their stage of software version were conducive for complete electronic transfer of treatment setup data. Therefore, we had processes in place that called for comprehensive reviews of manually entered setup parameters by an experienced medical physicist. Patients were treated on one of five Varian LINACs (three of which were equipped with multileaf collimators, (MLCs), or two MLC‐equipped Elekta LINACs. The Varian LINACs possess tertiary physical wedges and have the capability of delivering enhanced dynamic wedging. The Elekta machines use an internal universal wedge. The latter two wedge systems rely on external icons to assist with deciphering orientation on the treatment machine.

Our clinic tracks errors by means of a “notable event” procedure implemented by our Continuing Quality Improvement (CQI) Committee. Any events discovered mandate a physics review to determine dosimetric impact, if any, and are reported to the CQI Committee. The system is strategically designed to be nonpunitive to encourage disclosure of all events with a potentially negative consequence. Neither the individual(s) responsible for the event nor the individual(s) who missed catching the error are reprimanded to encourage reporting. Rather, information gleamed from the incident is used to modify procedures or to increase in‐services in the procedures where the error occurred. We examined events in the following treatment categories.

### A. External photon beam

For conventional photon beam treatment, we investigated errors of incorrect source‐to‐surface distance (SSD), incorrect energy, omitted wedge (physical, enhanced dynamic, or universal) or compensating filter, incorrect wedge or compensating filter orientation, and geometrical misses due to incorrect gantry, collimator or table angle, reversed field size, and setup errors. Because of specific incompatibilities between various systems, neither the Varis nor Desktop R&V systems possessed the capability of electronically verifying block or compensator trays or bolus. Many of these errors could initiate in either simulation, treatment planning, on the treatment machine, or somewhere in between.

### B. Electron beam

For electron beam therapy, the errors we investigated include the following: incorrect energy, incorrect SSD, and geometric misses. Electron beam therapy constitutes less than 5% of treatment beams used in our clinic.

### C. Special procedures

For special procedures we examined the impact of errors for total body irradiation (TBI), such as incorrect field size, dose rate, and treatment distance. The Varis R&V system interlocks field size by means of a special accessory mount. However, dose (or monitor unit (MU)) rate is not interlocked, and there is no practical method to electronically confirm the extended treatment distance because the treatment table is not used. For LINAC radiosurgery, we examined the impact of errors due to incorrect collimation setting and incorrect rotational parameters. IMRT was not included in this particular study.

### D. Error analysis

The likelihood of an error type was determined according to our history of detecting such errors. In addition, the longevity of the error propagating was evaluated for each error type. The impact of R&V along with our processes and procedures (pretreatment checks, port films, in vivo dosimetry) was examined. Dosimetric evaluation was conducted by using existing dosimetric data, treatment plans, or measurements. For various error types, anecdotal scenarios are detailed to decipher the pathway that led to such errors.

## III. RESULTS

We found geometric misses to have the highest error probability for both photons and for electrons. They most often occurred due to improper setup due to coordinate shift errors, incorrect field shaping, and reversed collimator jaws. The dosimetric impact is unique for each case and depends on the proportion of fields in error and volume mistreated. These errors were short‐lived due to rapid detection via port films. A summary of error types is found in Table 1. Examples of scenarios and pathways for some of the most common errors are presented below.

**Table 1:** Summary of errors over a 30‐month span. New patient starts are for 30‐month period. # of R&V were the number of errors felt to be potentially propagated by the R&V system.

Error type: typical # fractions: Method of detection	# of Events (% of starts)	# of R&V	Notes
incorrect treatment coordinate(s) : most often 1 fraction: port film	19 (0.48%)	7	*one case R& V not used.* Led to change in process whereby R&V display turned off in treatment room.
wrong gantry angle: most often 1 fraction: port film	15 (0.38%)	8	*one case R& V not used.* Led to change m process whereby R&V display turned off in treatment room.
wrong or omitted cerrobend block: most often 1 fraction: port film	15 (0.38%)	N/A	not part of R&V system
incorrect calculation: 2 to 5 fractions: diode leading or physics chart review	11 (0.28%)	N/A	not part of R&V system
wrong field size: one fraction: port film	9 (0.23%)	8	*one case affected dose p. q. enhanced dynamic wedge factor.* Led to change in process whereby R&V display turned off in treatment room.
incorrect collimator angle: one fraction: port film	8 (0.20%)	3	led to change in process whereby R&V display turned off in treatment room.
missing compensating filter: 2 to 5 fractions: diode of physics chart review	6 (0.15%)	N/A	
incorrect MU: 2 to 5 fractions: diode check	5 (0.13%)	4	*one case without R&V.* Led to change in override rights in MUs for therapists.
wrong photon energy: 2 to 3 fractions: diode check	3(<0.0%)	3	in‐service provided to therapists in reading treatment plans.
missing or incorrect MLC Shape: one fraction: port film	3(<0.0%)	3	
incorrect wedge direction: 1, 5, or 16 fractions: physics check, later diode check	3(<0.0%)	3	after second event: (1) diode readings taken off‐axis, (2) TP printout with rooms‐eye‐view placed in setup information.
incorrect number of fractions for given set of fields: 1 to 3 fractions: physics review	3(<0.0%)	2	
incorrect or rotated compensating filter: 2 or 11 fx fractions: therapist discovery for first case, diode check for second	2(<0.0%)	N/A	coinciding with wedge direction led action, diodes taken off‐axis.
patient treated head to gantry but scanned foot to gantry: one fraction: port film	1(<0.0%)	N/A	divergent wire built into immobilization system registration device.

### Scenario A: Shift error

During CT simulation, an initial reference is scribed in image planes denoting a projected isocenter. If subsequent treatment planning requires that the isocenter be placed 4.0 cm to the patient's left, requiring a need to shift the table lateral coordinates +4.0 cm, this will lead to the table coordinates to be used for treatments. However, due to a misinterpretation, a value of – 4.0 cm could used to determine the lateral table coordinate.

There is no feasible electronic mechanism to catch this error before treatment, short of having the coordinates checked by the physicists or therapist staff pretreatment, or a more dramatic solution of placing the patient on a conventional simulator to confirm the isocenter location. This particular error was determined to happen rarely enough (<0.5%) that our clinic continues to rely exclusively on the therapists visually checking the new isocenter against digitally reconstructed radiographs (DRRs) generated during virtual simulation or treatment planning. However, as we will discuss later, incorrect coordinate use, not necessarily due to shifts, is the most common error in our clinic. Related to these errors, the R&V system sometimes contained the incorrect coordinate information (vs. the paper chart) due to an incorrect shift instruction from the original reference point (projected isocenter) to the treatment isocenter. This led to a process change in regards to the R&V system, whereby the in‐room R&V display monitors were turned off. Although this may seem Draconian, we felt it was vital to emphasize that the R&V system was not a treatment setup system and should only be used to verify and record, maintaining the paper record as the primary setup documentation. The majority of shift errors occurred somewhat independent of the R&V system, whereby the resultant table coordinates in both the paper and electronic record were derived incorrectly. Therefore, prudent checks of the DRRs from treatment planning are the primary method of preventing such an error prior to obtaining a port film.

The pathway for such an error is as follows:

- Dosimetry staff incorrectly completes information sheet concerning shift. Incorrect table coordinates are calculated and entered. (Note: If the treatment plan was completed before treatment commenced and a direct DICOM transfer including table coordinates could be made, this error would be avoided.)
- Physics staff member fails to check shift instructions filled out by dosimetrist (reasonable process/training change).
- Therapist staff does not check DRR in room to ensure shift has been made correctly (definite process/training change).
- Diode does not facilitate error discovery.
- Port film taken and reviewed by physician who discovers error, which is corrected before subsequent fraction.

**Figure 1:**
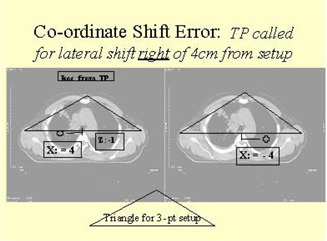
Visual description of how a coordinate shift error, in this case a lateral shift of –4 cm instead of the planned +4cm, would result in an incorrect isocenter placement.

A graphic depicting this scenario is exhibited in Fig. 1.

### Scenario B: Incorrect field shape

Multileaf collimator field shapes are generated for a patient requiring six fields that are planned for the treatment of prostate and seminal vesicles (PTV1) and an additional six smaller fields that are planned for the prostate only (PTV2). The MLC fields are exported to a directory for this patient for later “attachment” to the R&V system for treatment. However, the treatment planner responsible for setting up the treatment fields in the R&V system inadvertently assigns the smaller field shape to treat PTV1. Because the field shape differences are subtle (typically a 20% to 25% reduction in field area), a precheck of the field shapes for each field would be the only method of detecting such an error. However, since this error was determined to happen rarely (<0.1%), our clinic deemed this unnecessary.

The pathway for such an error is as follows:

- Dosimetry staff inadvertently attaches incorrect MLC shapes to fields. (Note: A direct DICOM transfer of TP data would have averted this error.)
- Physics staff checks individual R&V beam settings and MLC shape coinciding within jaw settings. However, since the initial and boost field shapes are similar and the boost shape is comfortably contained within initial jaw settings, the error is missed.
- Therapists do not identify subtle field shape differences seen in the DRRs and treat with small field shapes (reiteration of policy).
- Diode does not facilitate error discovery since $d_{\text{max}}$ doses are nearly the same for the field shapes.
- Physician discovers error on port film review.

The dosimetric impact is minimal because the reduced field shape is typically 20% smaller, and this type of error is discovered before second fraction is delivered.

### Scenario C: Reversed jaw settings

A patient receives simulation for opposed fields. The transverse jaw settings are asymmetric for the AP field $\left(X_{1}=8,X_{2}=4\right)$, which is the only field filmed on the simulator. When the chart is being prepared, the same jaw settings are used for each of the opposed fields (the opposed PA field should have used $X_{1}=4,X_{2}=8$). This scenario can also occur by a different mechanism due to nomenclature used in the treatment‐planning system, which depicts jaw settings according to direction from isocenter (left, right, upper, lower) and lists them transversely in reverse order for the jaw settings ($X_{2}$ followed by $X_{1}$). Even with these two scenarios, the error rate was still very low (<0.3%). This is due to the fact that treatment plans are completed and then reviewed as part of pretreatment checks by the experienced clinical physicist. The pathway and dosimetric error are similar to scenarios A and B.

For all of these scenarios, the portal film or portal image would assuredly catch these errors. However, if careful attention is not paid to the films, smaller errors, such as a 1 cm shift error, may not be immediately detected.

### A. Photon beam errors with dosimetric impact

#### Scenario D: Incorrect wedge direction

A four‐field technique is planned for a pelvis patient, including opposed 18 MV lateral beams requiring 60 wedges. The wedging is to be accomplished by use of enhanced dynamic wedging, which is accomplished by motion of collimating jaws while the beam is on and is limited to only one jaw pair (the upper Y jaws on Varian LINACs), and therefore only one plane of wedging is available unless a collimator rotation is applied. The collimator angle has to be chosen strategically. The chosen direction has notation according to which jaw is moving $\left(Y1_{\text{in}}\text{or}\;Y2_{\text{out}}\right)$ and is related to the “heel” end of the field. Because there is not an obvious method confirmation of the wedge orientation (heel) prior to treatment as is the case with physical wedges and incorrect collimator angle or choice of moving jaw (heel), an incorrect wedge direction could occur. There are external icons depicting the “heel” direction that are not as intuitive as physical wedges.

**Figure 2:**
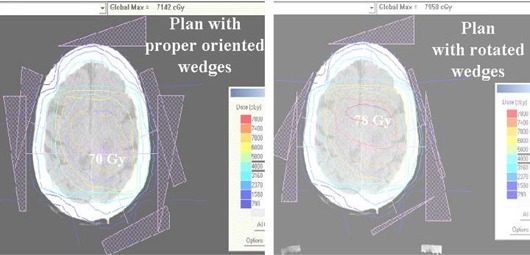
Comparison of isodoses for a central nervous system treatment plan that had called for seven wedged fields. The isodose map on the right demonstrates the resultant isodoses if the wedge orientation was reversed for four of the seven fields. The original plan used 6 MV photons with a target dose of 7000 cGy.

An additional problem with this type of virtual wedging is that the nomenclature used by treatment‐planning systems is not intuitive and not necessarily correlated to the nomenclature used by the treatment machines and/or R&V systems. Therefore, a misappropriated wedge direction could possibly go undetected by physics checks and by therapists on the first day of treatment. Unfortunately, in vivo dosimetry performed with a diode on central axis will not catch this particular error. Although we had the therapists check the final jaw position at the end of treatment (indicating the “toe” of the wedge), this policy was not carried out sufficiently to catch these errors. For the scenario described, the dosimetric error could be as high as 80% to a point 8 cm off‐axis, for opposed 60° wedged fields. We found this type of error occurred when the wedge orientation was not obvious in a 2D representation of the treatment, as is the case for central nervous system treatments requiring nonaxial fields. An example of a hypothetical intra‐cranial treatment with incorrect wedge directions is seen in Fig. 2. In this case, there are a total of seven beams for which the wedge was reversed from the intended orientations for four of the seven beams. This would have resulted in an increased high dose region (7800 cGy) outside of the tumor volume as compared with 7000 cGy in the correct plan.

The pathway for such an error is as follows:

- Dosimetry staff prepares intended plan for treatment based on MLC field shaping. After the planning is complete and the paper and electronic charts prepared, there is a decision to switch to cerrobend field shaping. In order to avoid having the weight of the block against the latch for some fields (a nonissue for MLC), the collimator is rotated 180° for these particular fields. Unfortunately, the original wedge direction is not corrected for the fields. (Mandated replanning would have avoided this error).
- Physicist checks individual R&V beam settings but does not detect the incorrect wedge direction.
- Therapists do not closely compare wedge directions as indicated on treatment plan and drawings with the icons on the treatment machine.
- Diode check does not facilitate error discovery as they are taken on central axis. (Change in procedure implemented, whereby diodes are now placed off‐axis. Therapists are instructed to position diode 2 cm to 3 cm toward the “heel” of the wedge as they perceive, but then denote the direction of the diode placement according to anatomical direction (i.e., for a breast patient with treated wedges, diode is placed anterior).
- Subsequent weekly review of chart by physicist catches the error.

#### Scenario E: Incorrect SSD

A breast cancer patient is planned for opposed tangents using 6 MV photons using a source‐to‐axis (SAD) technique (central axis SSD of 87 cm). After simulation and planning, potential for collision is detected at the first fraction. The chart is returned to dosimetry for recalculation for a 100 cm SSD technique and thus new collimator settings. However, the calculation is performed for the new field size, but the change to the new SSD is not clearly communicated. The patient is successfully treated with the new SSD of 100 cm. According to calculations using the SAD treatment MUs and the SSD treatment setup, an error of 26% dosage lower then prescribed occurs. Based on 100 cm SAD machines, a rule of thumb for dose difference for photons and electrons is a 2% change in dose per centimeter due to the inverse square effect.

The pathway for such an error is as follows:

- Dosimetry staff corrects paper and electronic chart for new field size and determines a new calculation is not needed.
- Chart bypasses physicist review after changes are made.
- Diode is taken, and upon review by the physicist it becomes obvious that the treatment SSD does not match the calculation SSD.

#### Scenario F: Incorrect photon energy

A physician initially prescribes 18 MV photons for a parallel‐opposed PA/AP fields to treat the pelvis. Calculations are performed. Due to a demanding load on the high‐energy machines, the patient is transferred to a single modality 6 MV LINAC because the PA separation (24 cm) is felt to not mandate use of high‐energy photons. However, no recalculation is performed, and MUs for an 18 MV beam are used. For most regions beyond the entrance region (first 3 cm from each surface), the dosimetric difference ranges from 6% to 16%. Larger differences occur in the buildup region.

The pathway for such an error is as follows:

- In haste, the patient is transferred to a low‐energy machine. Corrections are made notating new machine and energy, but the therapists fail to follow departmental procedures, and no new calculation is performed.
- Diode check reading catches error because the given doses (calculated 18 MV and measured 6 MV) are significantly different.

#### Scenario G: Incorrect electron energy

For electrons, the differences are more severe because the depth of penetration changes by approximately 0.5 cm/MeV. Therefore, the larger the energy difference, the larger the difference in dose delivered. In this example, a physician prescribes 16 MeV electrons to treat a soft tissue sarcoma to the depth of 4 cm (90% prescription), but due to a transposition error, the patient's machine MUs are calculated and subsequently treated with 6 MeV electrons. There is essentially no (<5% of prescription) dose delivered to the prescription depth in this case, although the prescription dose is delivered to the first 1.5 cm from the surface.

The pathway for such an error is as follows:

- Dosimetry staff inadvertently writes incorrect energy in paper chart. (Note: A direct DICOM transfer of TP data would avert this error.)
- Physicist misses the incorrect energy recording during initial chart review.
- During subsequent weekly chart checks, physics discovers discrepancy. (Note: Diode checks are not performed for electron beams.)
- Such errors emphasize the need to carefully review prescription parameters and subsequent recording of patients' treatment machine parameters.

#### B. Special procedure scenarios

For TBI, delivery errors were determined to be highly unlikely, with the exception of dose rate. This parameter and total treatment time (related to dose rate) are not checked by most R&V systems. However, for clinics employing low versus high (10 vs. 30cGy/cGy) dose rate protocols, such an error is possible. This occurs because some R&V systems do not use time or dose rate as a comparative fields due to variance of dose rate during the treatment. Therefore, the dose rate is appropriated by the MU/min rate set by the therapists. In our facility we employ low and high dose rates that are appropriate according to protocols that are either a single fraction of 550 cGy (high dose rate) or multifraction (low dose rate) with a typical 175 cGy per fraction. Therefore, the total time to deliver either treatment modality is ~18 min/fraction or ~9 min/field for our parallel opposed technique, although the MUs differ by a factor of 3. In one hypothetical case, if a high‐dose single fraction was to be delivered, but instead of appropriating the higher dose rate (according to a MU rate of 400MU/MU) a lower dose rate was set (by programming a low MU/min rate of 100), the patient could potentially be irradiated for as long as 50 min or 25min/min. This would be catastrophic for the patient if it were not for the ability to set a default maximum time to deliver a TBI treatment. However, a simple key function does allow the maximum time to be exceeded. In the opposite error scenario, whereby a low dose rate protocol was prescribed, the therapists could program a high dose rate, and a very rapid treatment (~2.5 min/field) could be delivered. In this case, the LINAC has no functionality to avoid such a mistreatment. Although this error is most likely less damaging to the patient, the biological impact is unknown.

Accelerator‐based stereotactic radiosurgery (SRS), typically not interlocked for field size, has the potential for delivering collimator field sizes that exceed the outer diameter of the stereotactic collimating system. The collimating systems have inner treatment diameters ranging from 4 mm to 40 mm and an outer diameter of 80 mm to 100 mm. This error would lead to significant dosage of the target region on the order of the prescriptive dose. This dosimetric impact is potentially serious because the MUs are well into the thousands. It is highly desirable that field sizes for LINAC SRS be either interlocked (not currently available on most LINACs) or integrated within an R&V system.

The two hypothetical scenarios clearly point out the need to have a rigorous double check policy (zero tolerance) in place for special procedures irradiation techniques.

#### C. Frequency analysis

From January 2001 to June 2003, all disclosed and/or discovered errors were tracked. Details were provided for each error with identification if the R&V system possibly propagated the error, or as in some cases, was not used for treatment. Throughout this period of time, decisions were made to change process or add an additional quality assurance check. These decisions were based on both frequency and dosimetric impact. Table 1 tallies the error types in order of frequency.

Other errors that occurred once and that did not lead to any policy or process change included the following (these are grouped according to error type, frequency, detection method, and R&V contribution):

- incorrect wedge angle: one fraction: diode detection; R&V propagated
- missing bolus: four fractions: physics check; not applicable
- incorrect electron energy: three fractions: physics check; R&V propagated
- missing wedge: two fractions: diode detected; R&V propagated
- incorrect table angle: one fraction: port film; R&V propagated
- one (of three fields) not treated: one fraction: therapist discovery; R&V not being used
- wrong patient parameters used for first of four fields: therapist discovery; R&V propagated

The error pathways can be categorized to three types:

1. An error that is easily detected after the first fraction by a port film. Although these are high in frequency, they are almost always detected before the second fraction and therefore have neither longevity nor subsequent dosimetric impact.
2. An error that is not detectable by port film but has a high likelihood of being detected by in vivo (diode) dosimeters and/or initial physicist chart review. These checks should take place before the second fraction but have gone as long as five fractions. In any case, there is minimal longevity and, therefore, only minimal dosimetric impact.
3. An error that is not detectable by port film or central axis diode or initial physicist review. These errors, although infrequent, have the chance to go undetected for many fractions and in many cases (e.g., an incorrectly oriented accessory) have very high dosimetric impact.

Breaking this up further according to dosimetric impact, longevity, and likelihood, Table 2 categorizes the errors previously mentioned for external beam even further.

**Table 2:** Error types from as categorized according to frequency, longevity, and dosimetric impact, using criteria below.

Error type	Frequency	Longevity	Dosimetric impact
incorrect treatment coordinate(s)	H	L	H
wrong gantry angle	H	L	M
wrong or omitted cerrobend block	H	L	L
incorrect calculation	H	M	M
wrong field size	M	L	L
incorrect collimator angle	M	L	M
missing compensating filter	M	M	M
incorrect MU	M	H	M
wrong photon energy	L	M	M
missing or incorrect MLC shape	L	L	L
incorrect wedge direction	L	H	H
incorrect number of fractious for given set of fields	L	M	M
incorrect or rotated compensating filter	L	M	M
patient treated head‐to‐gantry, scanned	L	L	H
FTG			

H=high;M=medium;L=low

Key:

Frequency: according to our history of occurrence rate

H:>0.25%

M:0.1% to 0.25%

L:<0.1%

Longevity: according to number of fractions

H: potentially >5 fractions and only picked up by careful weekly chart review

M: missed by port film, but assuredly discovered by diode on first check

L: caught by initial port film

Dosimetric impact: according to error of dose and/or volume

H: error of potentially >20% per fraction in terms of dose and/or treated volume

M: error between 10% and 20% per fraction in terms of dose and/or treated volume

L: error <10% per fraction in terms of dose and/or treated volume

**Figure 3:**
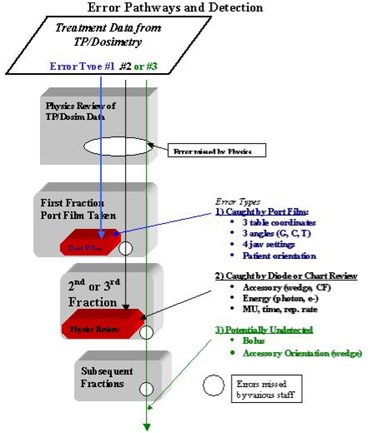
Flowchart of various error types. (1) Caught by port film review; (2) caught by chart and/or diode review; and (3) potentially undetected, and how the processes either stop or miss such errors.

Figure 3 graphically demonstrates the various pathways of the various error types.

**Figure 4:**
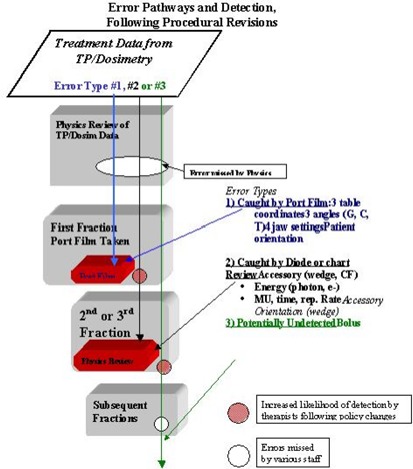
Flowchart of the same error types following changes made to policies and procedures, whereby certain errors were stopped earlier or were hampered from progressing.

In light of the errors with high frequency and of those with high dosimetric impact, we had made the following changes in our clinical process:

1. To reduce the errors with high frequency (H in Table 2), we determined that the R&V system had actually propagated some of these errors (Table 1). We therefore reduced override rights for the therapists along with reinforcing our departmental policy of reviewing the paper chart rather than the electronic R&V display. The therapists are not the originators of such errors, but these procedural changes certainly increase the likelihood of therapists catching these entry errors. In addition, we added a redundant system to confirm setup SSDs by having the therapists double‐check setup SSD, vertical table settings, and lateral setup marks on the patient. Some R&V systems do not allow finely tuned privileges for therapists and other users, which can compromise the effectiveness of the record and verify process. Figures 3 and 4 depict changes in error pathways before and after these changes.
2. To reduce the likelihood of the infrequent (low frequency) but dosimetrically significant error (high dosimetric impact and high longevity) of an incorrectly oriented accessory (wedge or compensation filter), our policy was changed to require diodes be placed off‐axis. This action requires the therapists to place the diode at a known distance off‐axis, at a known SSD. The therapist records the direction off‐axis relative to patient (e.g., anterior, superior, lateral). When the diode readings are reviewed, corrections are made for the off‐axis accessory transmission. Since this change was made, there have been multiple occasions in which the off‐axis diode reading has d etected an incorrect wedge orientation. Figures 3 and 4 depict changes for this particular error pathways before and after this change.

A revised error pathway chart (Fig. 4) demonstrates how some of these changes have altered potential paths. For example, by performing in vivo dosimetry off‐axis rather than on central axis, we essentially closed the pathway for the error to propagate beyond the second fraction, thereby reducing the longevity from high to medium.

## IV. CONCLUSIONS

To date, no one has studied the dosimetric impact of global errors in radiation oncology. Although there is heightened awareness that with the increase of automated devices there must be a parallel increase in quality check systems and processes, errors do and will continue to occur. This study has helped us identify and prioritize potential errors in our clinic according to frequency and dosimetric impact. For example, to reduce the use of an incorrect wedge direction, our clinic employs off‐axis in vivo dosimetry. To avoid a treatment distance setup error, we use both vertical table settings and optical distance indicator (SSD) values to properly setup fields. We strongly recommend that clinics focus their CQI efforts on errors that have high frequency and/or errors with high dosimetric impact and longevity and devise solutions to minimize such errors. As the R&V systems become more automated, there may be a positive and/or negative consequence. On the one hand, there will be increased desire for automation of patient setup and field appropriation (autofield sequencing). Simultaneously, these systems will also have higher degrees of connectivity with treatment planning and virtual simulation workstations via DICOM‐RT, thereby facilitating more accurate and efficient data transfer. This will require further error analyses.

Although the error scenarios and resolutions described in this paper are relevant mainly to our clinic, we feel many lessons can be extended to facilities, large and small. Our comprehensive in vivo dosimetry program can be implemented at any clinic. Bernier et al.^(^ ^10^ ^)^ describe an EORTC study of quality assurance programs that emphasizes the need for adequate staffing and training and wisely suggest plans for dummy runs for new procedures. Lanson et al.^(^ ^11^ ^)^ describe a very comprehensive in vivo dosimetry program that details the rationale for such a program. Finally, Brundage et al.^(^ ^7^ ^)^ describe an audit for a regional cancer center and how an audit system can analyze errors to find root causes. This is similar to our event‐reporting system, which has led to systematic changes.

Finally, we want to stress that the radiation oncology team must be constantly vigilant because no computer system can compensate for a team member's error in judgment, misunderstanding of physical concepts or technological limitations, or unsatisfactory planning and delivery of radiation therapy. We have begun examining potential errors in IMRT according to likelihood, pathways, and dosimetric impact, which we will report on at a later time.
